# *n*-Gram-Based Text Compression

**DOI:** 10.1155/2016/9483646

**Published:** 2016-11-14

**Authors:** Vu H. Nguyen, Hien T. Nguyen, Hieu N. Duong, Vaclav Snasel

**Affiliations:** ^1^Faculty of Information Technology, Ton Duc Thang University, Ho Chi Minh City, Vietnam; ^2^Faculty of Computer Science and Engineering, Ho Chi Minh City University of Technology, Ho Chi Minh City, Vietnam; ^3^Faculty of Electrical Engineering and Computer Science, VSB-Technical University of Ostrava, Ostrava, Czech Republic

## Abstract

We propose an efficient method for compressing Vietnamese text using* n*-gram dictionaries. It has a significant compression ratio in comparison with those of state-of-the-art methods on the same dataset. Given a text, first, the proposed method splits it into* n*-grams and then encodes them based on* n*-gram dictionaries. In the encoding phase, we use a sliding window with a size that ranges from bigram to five grams to obtain the best encoding stream. Each* n*-gram is encoded by two to four bytes accordingly based on its corresponding* n*-gram dictionary. We collected 2.5 GB text corpus from some Vietnamese news agencies to build* n*-gram dictionaries from unigram to five grams and achieve dictionaries with a size of 12 GB in total. In order to evaluate our method, we collected a testing set of 10 different text files with different sizes. The experimental results indicate that our method achieves compression ratio around 90% and outperforms state-of-the-art methods.

## 1. Introduction

According to [1], data compression is a process of converting an input data stream into another data stream that has a smaller size. A stream can be a file, a buffer in memory, or individual bits sent on a communications channel. The main objectives of data compression are to reduce the size of input stream and increase the transfer rate as well as save storage space. Typically, data compression techniques are classified into two classes, that is, lossless and lossy, based on the result of the decompression phase.

Text compression is a field of data compression, which uses the lossless compression technique to convert an input file to another form of data file. It cannot use the lossy compression technique because it needs to recover the exact original file from the compressed file. If lossy compression technique was used, the meaning of the decompression file will be different from the original file. Several techniques have been proposed for text compression in recent years. Most of them are based on the same principle of removing or reducing redundancies from the original input text file. The redundancy can appear at character, syllable, or word levels. This principle proposed a mechanism for text compression by assigning short codes to common parts, that is, characters, syllables, words, or sentences, and long codes to rare parts.

In recent years, several techniques have been developed for text compression. These techniques can be further classified into four major types, that is, substitution, statistical, dictionary, and context-based method. The substitution text compression techniques replace a certain longer repetition of characters with a shorter one. A technique that is a representative of these techniques is run-length encoding [2]. The statistical techniques usually calculate the probability of characters to generate the shortest average code length, such as Shannon-Fano coding [3, 4], Huffman coding [5], and arithmetic coding [6, 7]. The next type consists of dictionary techniques, which involve substitution of a substring of text by an index or a pointer code. They relate to a position in the dictionary of the substring. Representatives of these techniques are LZW [8], LZ77 [9], and LZ78 [10]. The last type is context-based techniques, which involve the use of minimal prior assumptions about the statistics of the text. Normally, they use the context of the text being encoded and the history of the text to provide more efficient compression. Representatives of this type are Prediction by Partial Matching (PPM) [11] and Burrow–Wheeler transform (BWT) [12]. Every method has its own advantages and disadvantages when being applied in a specific field. None of the above methods has been able to achieve the best results in terms of compression ratio.

Normally, users will decide to choose the appropriate method based on their purposes. With systems that allow the reconstruction of information from the output not to be exactly the same as the input, we can use lossy methods, such as systems to compress images and voice messages. With systems that require the original data to be recovered exactly from the compressed data, we must use lossless methods, such as text compression systems.

This paper presents the first attempt at text compression using* n*-gram dictionaries, and the contribution has three attributes; that is, (1) it is a method for text compression using* n*-gram dictionaries, (2) it collects the text corpus of the Vietnamese language from the Internet and builds five* n*-gram dictionaries with nearly 500,000,000* n*-grams, and (3) a test set of 10 different text files with different sizes to evaluate our new system and compare it with my two previous methods [13, 14] and also with other methods. The rest of this paper is organized as follows.  Section 2 presents earlier work related to this effort.  Section 3 presents our proposed method, and Section 4 presents our experiments and results. Our conclusions are presented in Section 5.

## 2. Related Work

In recent years, most text compression techniques have been based on dictionary, word, or character levels [15–18]. Reference [15] proposed a method to convert the characters in the source file to a binary code, where the most common characters in the file have the shortest binary codes and the least common have the longest. The binary codes are generated based on the estimated probability of the character within the file and are compressed using 8-bit character word length. In [16], the authors proposed a method that combined word with LZW. First, their method splits input text to word and nonword and then uses them as initial alphabet of LZW. Reference [17] proposed a technique to compress short text messages based on two phases. In the first phase, it converts the input text consisting of letters, numbers, spaces, and punctuation marks commonly used in English writing to a format which can be compressed in the second phase. In the second phase, it proposes a transformation which reduces the size of the message by a fixed fraction of its original size. In [18], the authors proposed a word-based compression variant based on the LZ77 algorithm and proposed and implemented various ways of sliding windows and various possibilities of output encoding. In a comparison with other word-based methods, their proposed method is the best. In these research, they do not consider the structure of words or morphemes in the text.

In addition, there are some approaches to text compression based on syllables, BWT. These approaches involve some languages that have morphology in the structure of words or morphemes (e.g., German, Arabic, Turkish, and Czech) such as in [19–23]. Reference [19] presented a new lossless text compression technique which utilizes syllable-based morphology of multisyllabic languages. The proposed method is designed to partition words into its syllables and then to produce their shorter bit representations for compression. The number of bits in coding syllables depends on the number of entries in the dictionary file. In [20], the authors proposed a genetic algorithm in syllable-based text compression. This algorithm was used to determine for the characteristic of syllables. These characteristics are stored into dictionary, which is part of the compression algorithm and it is not necessary to place them into compressed data. This leads to reduction of used space. In [21, 22], Lansky and his colleagues were the first to propose a method for syllable-based text compression techniques. In their papers, they focused on specification of syllables, methods for decomposition of words into syllables, and using syllable-based compression in combination of the principles of LZW and Huffman coding. In [23], the authors first proposed a method for small text file compression based on the Burrow–Wheeler transformation. This method combines the Burrow–Wheeler transform with the Boolean minimization at the same time.

In our previous papers for Vietnamese text compression [13, 14], we proposed a syllable-based method based on morphology and syllable dictionaries in [13]. With each morphosyllable, it is split into a consonant and a syllable, and they are compressed based on their corresponding dictionaries. This method has a compression ratio that converges to around 73%, and it is suitable for small text files. The method in [14] compressed text based on a trigram model; it splits a text file into trigrams and compresses these trigrams using a trigrams dictionary. This method achieves an encouraging compression ratio around 83%.

## 3. Proposed Method

**Figure 1:**
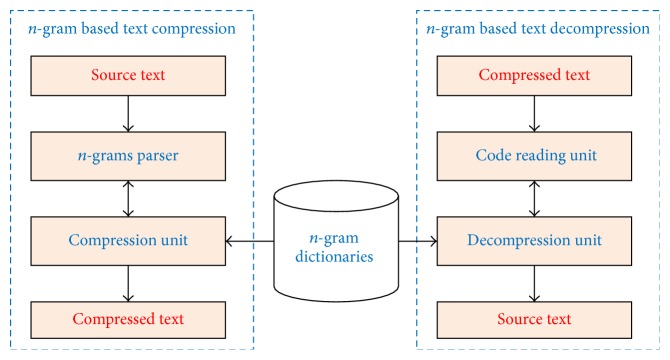
*n*-gram-based text compression model.

In this section, we present a method for Vietnamese text compression using* n*-gram dictionaries. This model has two main modules. The first module is used for text compression and the second module performs decompression.  Figure 1 describes our text compression model. In our model, we use* n*-gram dictionaries for both compression and decompression. We will describe the model in detail in the following subsections.

### 3.1. *n*-Gram Theory and Dictionaries

#### 3.1.1. *n*-Gram Theory

In this paper, we use* n*-gram theory mentioned from [24]: an* n*-gram is a contiguous sequence of *n* items from a given sequence of a text or speech. An item can be a phoneme, a syllable, a letter, a word, or a morphosyllable. In general, an item is considered as an atomic unit. An* n*-gram of one item, two items, or three items is referred to as a “unigram,” a “bigram,” or a “trigram,” respectively. Larger sizes are sometimes referred to by the number of items* n*, for example, “four-gram” and “five-gram.”

#### 3.1.2. Dictionaries

Since we focus on Vietnamese, we build five different Vietnamese dictionaries of unigram, bigram, trigram, four grams, and five grams corresponding to the number of grams compressed.  Table 1 shows these dictionaries with their number of* n*-grams and size. These dictionaries have been built based on a text corpus collected from the Internet. The size of the text corpus is around 2.5 GB. We use SRILM (http://www.speech.sri.com/projects/srilm/) to generate* n*-grams for these dictionaries. To increase the speed of searching in these dictionaries, we arranged them according to the alphabet.  Table 1 describes the size and number of* n*-grams in each dictionary.

### 3.2. Compression

As presented in Figure 1, the compression module takes a source text as an input and then passes the text through two submodules, that is,* n*-grams parser and compression unit, to compress it. In following subsections, we explain in detail.

#### 3.2.1. *n*-Gram Parser

*n*-gram parser has been used to read a source text file, splits it to sentences based on newline, and reads the number of grams in the combination with the result of the compression unit. In* n*-gram parser, we use five kinds of* n*-gram to store for unigram, bigram, trigram, four grams, and five grams. Based on the result of the compression unit, the* n*-gram parser decides how many grams will be read next.  Algorithm 1 shows the pseudocode of this phase. If five grams was found in the five-gram dictionary, that is, index > 0, the force_four_gram_compression function would be called to encode all previous* n*-grams (unigram, bigram, trigram, and four grams); then the compress function would be called to encode this five grams. Next, the* n*-gram parser reads next five grams in the input string. Otherwise, it would split one leftmost gram of five grams for four grams and read one gram more from the input string for five grams. When the number of grams of four-gram was 4, it calls the four_gram_compression function.

Algorithm 2 shows the pseudocode of the four_gram_compression function. This function is used to compress four grams if it occurs in four-gram dictionary. Otherwise, it splits one leftmost gram of the four-gram variable for the trigram variable. Similar to this function, we have the trigram_compression, the bigram_compression, and the unigram_compression function.

The force_four_gram_compression is called to encode all four-gram, trigram, bigram, and unigram when five-gram variable is found in the five-gram dictionary. Similar to this function, we have the force_trigram_compression, the force_bigram_compression, and the force_unigram_compression function (Algorithm 3).

#### 3.2.2. Compression Unit

The compression unit uses the result from the* n*-gram parser to decide how many grams will be compressed and what kind of* n*-gram dictionaries should be used. Based on the number of* n*-grams in each dictionary, we will construct the number of bytes to encode each* n*-gram corresponding to the dictionary.  Table 2 describes the number of bytes used to encode each* n*-gram of each dictionary.

To classify the dictionary that was used to encode each* n*-gram and the other cases, we use three most significant bits (MSB) of the first byte of each encoded byte.  Table 3 describes the value of these bits corresponding to each dictionary.

The index of each* n*-gram corresponding to each dictionary is encoded in the bits after the first three bits of the first byte. As seen in Table 3, there are two special cases for the* n*-gram dictionary: a newline and a unigram that does not appear in the unigram dictionary corresponding to a value of “newline” and “others.” In these cases, the compression unit will encode as follows:

- When the result received from the* n*-gram parser is the newline, the compression unit will encode the value “110” for the first three bits of MSB, and the next five bits of this byte will have the value “00000.”
- When the result is the others, the three MSB of the first byte are “111” and the next five bits of this byte present the number of bytes which were used to encode this gram.

### 3.3. Decompression

As seen in Figure 1, the decompression module takes a compressed text as an input and then passes the text through two submodules, that is, code reading unit and decompression unit, to decompress it. We explain in detail in following subsections.

#### 3.3.1. Code Reading Unit

First, this unit reads the compressed text from the compression phase. This result becomes the input sequence of the code reading unit. The code reading unit splits this input sequence byte to byte. Then, it reads the first byte of the input sequence and splits and analyzes the first three bits of this byte to classify the dictionary to which this* n*-gram belongs. Based on this result, this unit will read more bytes from the input sequence.  Table 2 shows the number of bytes that the code reading unit reads after the first byte according to the classification of the dictionary. After reading these bytes, it transfers them to the decompression unit and repeats its work until the input sequence is null.

#### 3.3.2. Decompression Unit

This unit receives the results from the code reading unit. It decodes these results according to the classification of the dictionary as follows.

- Decode* n*-grams occurring in dictionaries
  1. Identifying the dictionary: based on the classification dictionary from the code reading unit
  2. Identifying the index of an* n*-gram in the dictionary: based on the value calculated from bytes that were read by the code reading unit
  3. Decode* n*-gram: when the classification of the dictionary has a value from one to five, the decompression unit decodes the* n*-gram in the dictionary based on the index of the* n*-gram
- Decode* n*-grams that do not occur in dictionaries
  1. Decode newline: when the classification of dictionary is a “newline,” it means that the value of the first three bits is 110. The decompression unit decodes a newline for this* n*-gram
  2. Decode others: when the classification of the dictionary is “others,” based on the value of the remaining bits of the first byte, the decompression unit will decode all bytes after the first byte

After finishing the decoding for one* n*-gram or other cases, the decompression unit reads the next result from the code reading unit and repeats the decompression tasks to decode other* n*-grams or other cases until it reads the last byte.  Algorithm 4 shows the pseudocode of the decompression phase.

### 3.4. Compression Ratio

Compression ratio is used to measure the efficiency of the compression method. The stronger the compression ratio is, the better the quality of this method is. The compression ratio can be calculated by

(1) $$\begin{array}{c} CR=\left(\;1-\frac{compressed\_file\_size}{original\_file\_size}\;\right)\times 100, \end{array}$$

where original_file_size is size of the original file and compressed_file_size is size of the compressed file.

### 3.5. The Complexity of Our Method

Let *n* be the number of* n*-grams in the source text and* a*,* b*,* c*,* d*, and *e* be the number of five grams, four grams, trigrams, bigrams, and unigrams, respectively, in dictionaries. Let *k* be log_2_(*a*) + log_2_(*b*) + log_2_(*c*) + log_2_(*d*) + log_2_(*e*). According to pseudocode from Algorithm 1, in the worst case, all five grams, four grams, trigrams, and bigrams do not occur in five grams, four grams, trigram, and bigram dictionary, respectively. Hence, the complexity of our method is *O*(*k∗n*).

### 3.6. Example

#### 3.6.1. Compression Phase

Let us encode the following sequence using the* n*-gram approach.

*Nén dữ liệu nhằm giảm kích thước dữ liệu để tăng tốc độ truyền cũng như tiết kiệm không gian lưu trữ*

Assume that we have five dictionaries for unigram, bigram, trigram, four grams, and five grams, as seen in Table 4.

The* n*-gram parser first encounters the first five-gram* Nén dữ liệu nhằm giảm* and copies it to the five-gram variable. This pattern is not in the five-gram dictionary, so it splits the first gram of this pattern for the four-gram variable and concatenates the next gram of the input sequence to the five-gram variable. The content of the five-gram and four-gram variables becomes* dữ liệu nhằm giảm kích* and* Nén*, respectively. Then, it checks the number of grams in the four-gram variable, which is one at this time. In this case, the value is less than four; it bypasses the four_gram_compression and turns back to the five-gram variable. Because this pattern is not in the five-gram dictionary, similar to the first case, it splits the first gram of this five-gram to the four-gram variable and concatenates the next gram of the input sequence to the five-gram variable. The content of the five-gram and four-gram variables shall become* liệu nhằm giảm kích thước* and* Nén dữ*, respectively. Then, it checks the number of grams in the four-gram variable, which is two now. This value is less than four, similar to the first case; it turns back to five-gram variable. It repeats these operations until the content of the five-gram variable is* nhằm giảm kích thước dữ* and the four-gram variable is* Nén dữ liệu*. This five-gram pattern is not in five-gram dictionary, so it splits the first gram of this pattern for the four-gram variable and concatenates the next gram of the input sequence to the five-gram variable. The content of the five-gram and four-gram variables shall become* giảm kích thước dữ liệu* and* Nén dữ liệu nhằm*, respectively. It checks the number of grams in the four-gram variable, which is four now. It calls the four_gram_compression as presented in Algorithm 2. The four_gram_compression searches the four-gram pattern in the four-gram dictionary, which is not found in the four-gram dictionary. It splits the first gram of this pattern into the trigram variable. The content of the four-gram and the trigram variable becomes* dữ liệu nhằm* and* Nén*, respectively. Then, it checks the number of grams in the trigram variable, which is one at this time. So, it bypasses the trigram_compression, exits the four_gram_compression, and turns back to five-gram variable in Algorithm 1. The first five steps as seen in Table 5 show the content of the five-gram, four-gram, and trigram variables throughout these steps.

At Step  6, first, the* n*-gram parser checks the value of the five-gram variable in the five-gram dictionary. This pattern is in the dictionary; therefore, it calls the compression unit to encode all bigram, trigram, and four grams. Then, it encodes the five-gram. When the compression unit is finished, the* n*-gram parser reads the next five grams from the input sequence. In Table 5, Steps  6.1 to 6.4 show all substeps of Step  6 and in Table 6, Steps  6.2 to 6.4 show the encoder output sequence.

As seen in Table 5, at Step  6.1, the* n*-gram parser splits the first gram of the four-gram variable for the trigram variable, and the content of the four-gram and trigram variable shall become* liệu nhằm* and* Nén dữ*, respectively. Then, it checks the number of grams in the trigram variable, which is two at this time. So, it bypasses the trigram_compression and moves to Step  6.2. At Step  6.2, it continues splitting the first gram of the four-gram variable for the trigram variable. The content of the four-gram and trigram variables shall become* nhằm* and* Nén dữ liệu*, respectively. Next, it checks the number of grams in the trigram variable, which is three at this time. It then searches for this trigram in the trigram dictionary. Because this trigram is in the trigram dictionary, it calls the compression unit to encode bigram in the bigram variable. In this case, the bigram variable is null. It calls the compression unit to encode the trigram in the trigram variable and moves to the next substep. The encoded sequence of this trigram is shown in Table 6 at Step  6.2. The first three bits of this encoded sequence which have value* 011* refer to trigram dictionary as seen in Table 3 and all remaining bits refer to the index of this trigram in the trigram dictionary.

At Step  6.3, the bigram and trigram variables are null; it counts the number of grams in the four-gram variable, which is 1 in this case; then it copies this gram to the unigram and searches for this unigram in the unigram dictionary. This unigram is in dictionary so it calls the compression unit to encode this unigram. The encoder output sequence of this unigram is shown in Table 6 at Step  6.3. At Step  6.4, it calls the compression unit to encode the five-gram in the five-gram variable, and the encoder output sequence of this five-gram is shown in Table 6 at Step  6.4. Then it reads the next five-gram in the input sequence to the five-gram variable. At this time, the content of the five-gram variable is* để tăng tốc độ truyền*.

The* n*-gram parser and the compression unit will process similar to previous cases for all remaining grams of the input sequence. The results of these steps are shown in Table 5 from Step  7 to Step  13.4. The encoder output sequences are shown in Table 6 from Step  7 to Step  13.4. The final encoder output sequence is the result of concatenation of all encoder output sequences from Step  6.1 to 13.4 in Table 6. The final encoder output sequence is

*01100000000000000000000000000001|0010000000000001|10100000000000000000000000000001*
*|10100000000000000000000000000010|01000000000000000000000000000001|1000000000000000*
*0000000000000001|0010000000000010|0010000000000011*.

#### 3.6.2. Decompression Phase

In this section, the encoder output sequence from the previous example is taken and is decoded using the decompression unit. The encoder output sequence in the previous example was

*01100000000000000000000000000001|0010000000000001|10100000000000000000000000000001*
*|10100000000000000000000000000010|01000000000000000000000000000001|1000000000000000*
*0000000000000001|0010000000000010|0010000000000011*.

The decompression unit uses the same dictionaries as the compression unit as seen in Table 4. It reads the first byte of the input sequence; the content of this first byte is* 01100000*. The first three bits are split, and the value of these three bits is* 011*. It finds the corresponding* n*-gram dictionary of these three bits and the number of bytes that is read more as presented in Table 3. In this case, the* n*-gram dictionary is the trigram dictionary and the number of bytes that is read more is 3. The decoder reads the next three bytes from the input sequence. The index of the entry was calculated based on the value of all remaining bits after the first three bits and the three bytes that is read more. The entry is determined based on this index. The decoder repeats these steps until it reads the last byte of the input sequence.  Table 7 shows all steps and results of the decompression phase.

The final decoder output sequence is the result of concatenation of all decoder output sequences from Step  1 to Step  8 as presented in Table 7. With each decoder output sequence from Step  1 to Step  7, we add one space character before the concatenation. The final encoder output sequence is* Nén dữ liệu nhằm giảm kích thước dữ liệu để tăng tốc độ truyền cũng như tiết kiệm không gian lưu trữ*.

## 4. Experiments

We conducted an experiment to evaluate our method, using a dataset that is randomized collection from some Vietnamese news agencies. The dataset includes 10 files completely different in size and content.

**Figure 2:**
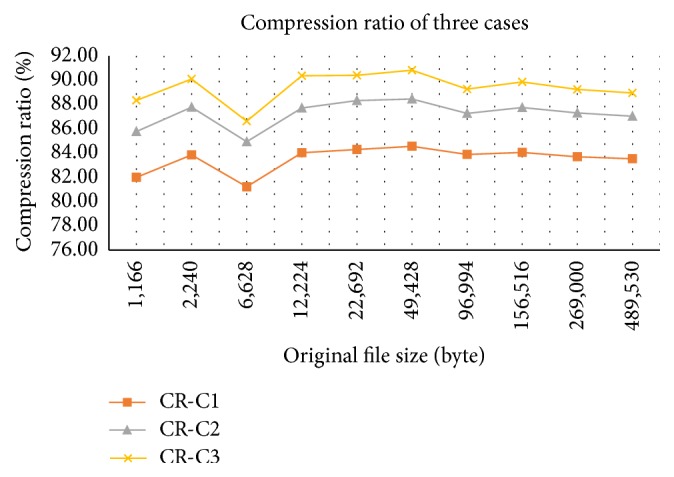
Comparison between the three cases.

**Figure 3:**
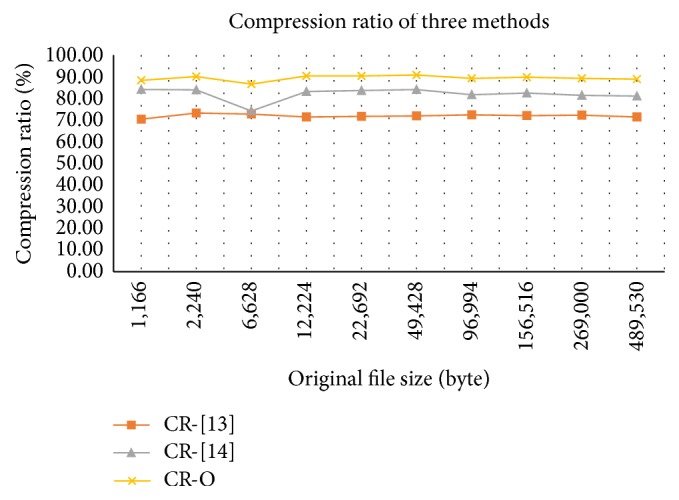
Compression ratio of our method [13, 14].

**Figure 4:**
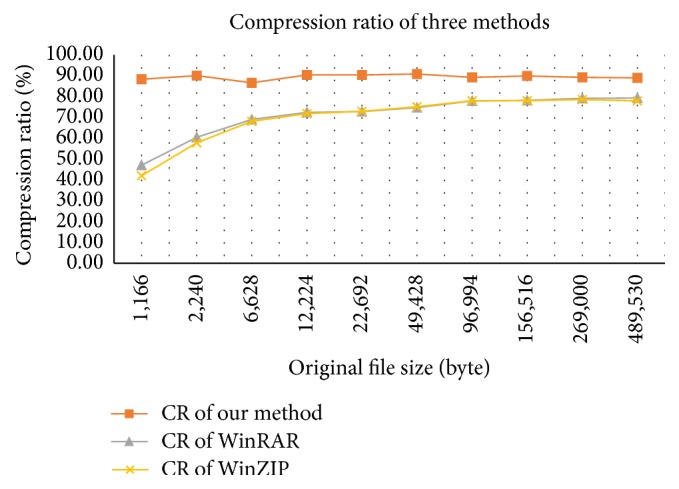
Compression ratio of our method, WinRAR, and WinZIP.

**Figure 5:**
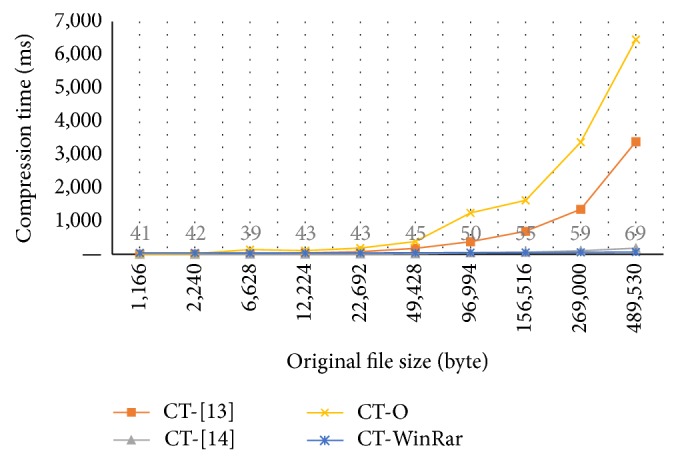
Compression time of four methods.

**Figure 6:**
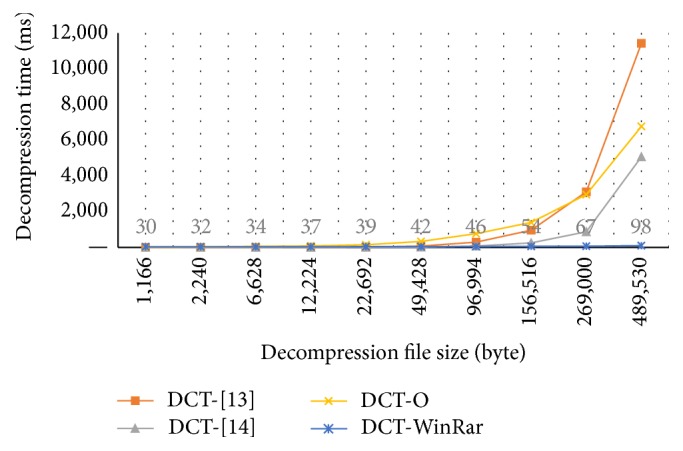
Decompression time of four methods.

In order to evaluate the effects of a combination of various* n*-gram dictionaries, we conducted three experiments with three kinds of systems. In the first case, we build a system with unigram, bigram, and trigram dictionaries. Next, we extend the first one with four-gram dictionary. Lastly, we extend the second one with five-gram dictionary. The results of the three experiments are shown in Table 8. As presented in Table 8, we find out that the compression ratio from the third case is the best, follow-up is the second case, and the last one comes from the first case. The compression ratio in this section was used according to (1). In Tables 8, 9, and 10 and Figures 2, 3, 4, 5, and 6, we have some abbreviations and meanings as follows: OFS: original file size in byte; CFS: compressed file size in byte; CR: compression ratio; C1, C2, and C3: three cases above, respectively; O: our method; RAR: WinRAR; ZIP: WinZIP.

As seen in Figure 2, the compression ratio when we combine all five dictionaries is the highest.

In order to evaluate our method with the methods presented in [13, 14], we compress the input files using these methods. In Table 9, we show the results of the current method in 10 cases in comparison with the methods in [13, 14]. As shown in Table 9 and Figure 3, the compression ratio of our method is better than the methods presented in [13, 14] for any size of text in our test cases.

Table 10 and Figure 4 show the results of our method in comparison with those of other methods, such as WinZIP version 19.5 (http://www.winzip.com/win/en/index.htm), the software combining LZ77 [8] and Huffman coding, and WinRAR version 5.21 (http://www.rarlab.com/download.htm), the software combining LZSS [25] and Prediction by Partial Matching [11]. The experimental results show that our method achieves the highest compression ratio on the same testing set.

Tables 11 and 12 and Figures 5 and 6 show the compression and decompression time of our method in comparison with the methods in [13, 14] and WinRAR, respectively. In these tables and figures, we have some abbreviations and meanings as follows: CT: compression time; DT: decompression time; RAR: WinRAR; O: our method; ms: millisecond.

As presented in Table 12 and Figure 5, the compression time of our method is higher than those of other methods.

As presented in Table 12 and Figure 6, the decompression time of our method is higher than [14] but it is slower than [13] and WinRAR.

## 5. Conclusions

In this paper, we present a novel method using* n*-gram dictionaries for text compression. We build five different* n*-gram dictionaries range from unigram to five grams from a 2.5 GB text corpus and obtain approximately 12 GB* n*-grams. We conduct experiments on a dataset of 10 files with different sizes and content in three different scenarios. The first scenario uses unigram, bigram, and trigram dictionaries. The second scenario extends the first one with four-gram dictionary and the final scenario extends the second one with five-gram dictionary. The experimental results show that our method achieves the performance comparable with those of state-of-the-art methods including WinZIP and WinRAR in terms of compression ratio, while it is slower than these two of WinZIP and WinRAR. Speeding-up looking-up process of dictionaries may lead to foster the running time of ours method. We put this perspective as a direction of research in future.

## Figures and Tables

**Algorithm 1:**
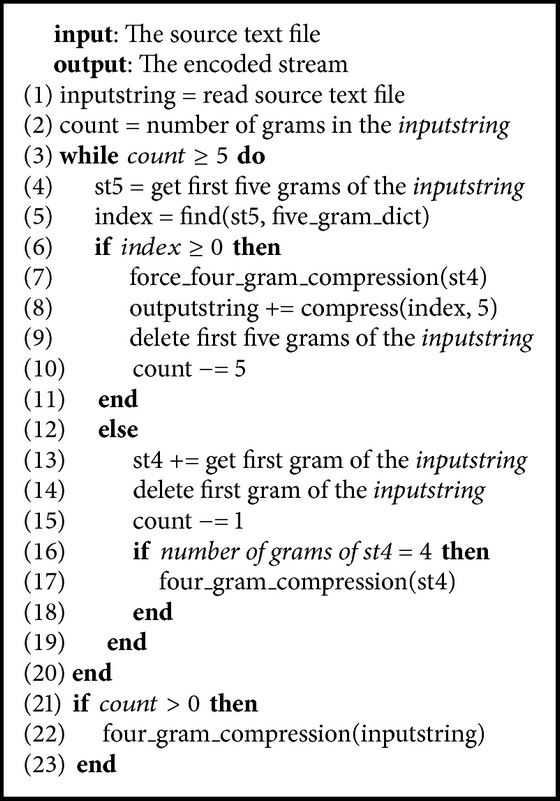
Pseudocode of the compression phase.

**Algorithm 2:**
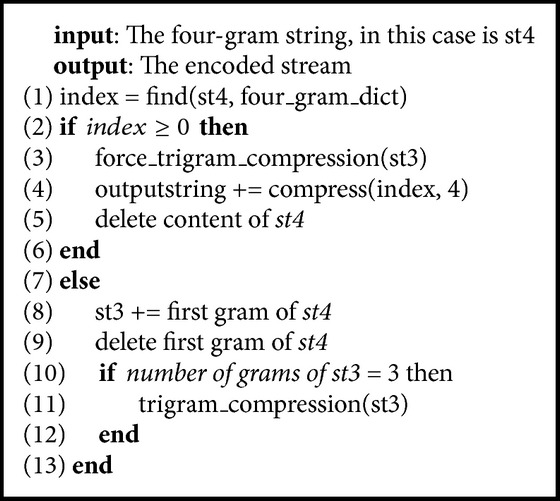
Pseudocode of the four_gram_compression.

**Algorithm 3:**
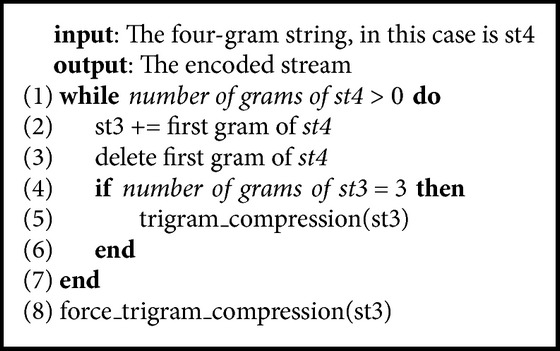
Pseudocode of the force_four_gram_compression.

**Algorithm 4:**
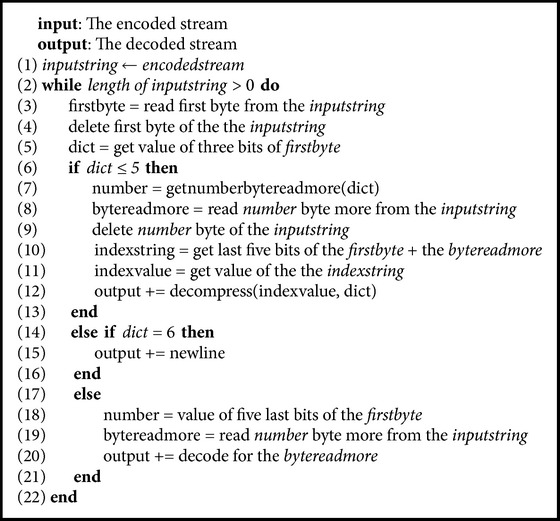
Pseudocode of the decompression phase.

**Table 1:** *n*-gram dictionaries.

*n*-gram dictionary	Number of *n*-grams	Size (MB)
1	7,353	0.05
2	20,498,455	474
3	84,003,322	1,586
4	169,916,000	4,155
5	225,203,959	6,800

**Table 2:** Number of encoded bytes for each *n*-gram of each dictionary.

*n*-gram dictionary	Number of *n*-grams	Number of bytes
1	7,353	2
2	20,498,455	4
3	84,003,322	4
4	169,916,000	4
5	225,203,959	4

**Table 3:** Value of three MSB and number of bytes.

*n*-gram dictionary	Value of three MSB	Number of bytes is read more
1	0 0 1	1
2	0 1 0	3
3	0 1 1	3
4	1 0 0	3
5	1 0 1	3
Newline	1 1 0	0
Others	1 1 1	Value of five bits after three first bits of current byte

**Table:** (a) Unigram dictionary

Index	Entry
1	nhằm
2	lưu
3	trữ

**Table:** (b) Bigram dictionary

Index	Entry
1	cũng như

**Table:** (c) Trigram dictionary

Index	Entry
1	Nén dữ liệu

**Table:** (d) Four-gram dictionary

Index	Entry
1	tiết kiệm không gian

**Table:** (e) Five-gram dictionary

Index	Entry
1	giảm kích thước dữ liệu
2	để tăng tốc độ truyền

**Table 5:** All steps and values of *n*-grams.

Step	Five-gram variable	Four-gram variable	Trigram variable	Bigram variable
1	Nén dữ liệu nhằm giảm			
2	dữ liệu nhằm giảm kích	Nén		
3	liệu nhằm giảm kích thước	Nén dữ		
4	nhằm giảm kích thước dữ	Nén dữ liệu		
5	giảm kích thước dữ liệu	dữ liệu nhằm	Nén	
6.1	giảm kích thước dữ liệu	liệu nhằm	Nén dữ	
6.2	giảm kích thước dữ liệu	nhằm		
6.3	giảm kích thước dữ liệu			
6.4	để tăng tốc độ truyền			
7	cũng như tiết kiệm không			
8	như tiết kiệm không gian	cũng		
9	tiết kiệm không gian lưu	cũng như		
10	kiệm không gian lưu trữ	cũng như tiết		
11	không gian lưu trữ	như tiết kiệm	cũng	
12	gian lưu trữ	tiết kiệm không	cũng như	
13.1	lưu trữ	tiết kiệm không gian		
13.2				lưu trữ
13.3				trữ
13.4				

**Table 6:** Encoder output sequences.

Step	Encoding of dictionary	Encoded sequence
6.2	011	00000000000000000000000000001
6.3	001	0000000000001
6.4	101	00000000000000000000000000001
7	101	00000000000000000000000000010
13.1	010	00000000000000000000000000001
13.2	100	00000000000000000000000000001
13.3	001	0000000000010
13.4	001	0000000000011

**Table 7:** All steps and the results of the decompression phase.

Step	First byte	Dict. nbm bits to calculate index			Index value	Decoder output sequence
1	01100000	011	3	00000000000000000000000000001	1	Nén dữ liệu
2	00100000	001	1	0000000000001	1	nhằm
3	10100000	101	3	00000000000000000000000000001	1	giảm kích thước dữ liệu
4	10100000	101	3	00000000000000000000000000010	2	để tăng tốc độ truyền
5	01000000	010	3	00000000000000000000000000001	1	cũng như
6	10000000	100	3	00000000000000000000000000001	1	tiết kiệm không gian
7	00100000	001	1	0000000000010	2	lưu
8	00100000	001	1	0000000000011	3	trữ

**Table 8:** Compression ratio of three experience cases.

Number	OFS	CFS-C1	CR-C1	CFS-C2	CR-C2	CFS-C3	CR-C3
1	1,166	210	81.99%	166	85.76%	136	88.34%
2	2,240	362	83.84%	274	87.77%	222	90.09%
3	6,628	1,245	81.22%	999	84.93%	887	86.62%
4	12,224	1,954	84.02%	1,503	87.70%	1,179	90.36%
5	22,692	3,565	84.29%	2,652	88.31%	2,180	90.39%
6	49,428	7,638	84.55%	5,712	88.44%	4,538	90.82%
7	96,994	15,636	83.88%	12,359	87.26%	10,416	89.26%
8	156,516	24,974	84.04%	19,188	87.74%	15,889	89.85%
9	269,000	43,887	83.69%	34,182	87.29%	28,937	89.24%
10	489,530	80,685	83.52%	63,472	87.03%	54,117	88.95%

**Table 9:** CR of the current method with the methods presented in [13, 14].

Number	OFS	CFS-[13]	CR-[13]	CFS-[14]	CR-[14]	CFS-O	CR-O
1	1,166	345	70.41%	185	84.13%	136	88.34%
2	2,240	599	73.26%	359	83.97%	222	90.09%
3	6,628	1,803	72.80%	1,710	74.20%	887	86.62%
4	12,224	3,495	71.41%	2,057	83.17%	1,179	90.36%
5	22,692	6,418	71.72%	3,702	83.69%	2,180	90.39%
6	49,428	13,881	71.92%	7,870	84.08%	4,538	90.82%
7	96,994	26,772	72.40%	17,723	81.73%	10,416	89.26%
8	156,516	43,701	72.08%	27,434	82.47%	15,889	89.85%
9	269,000	74,504	72.30%	49,902	81.45%	28,937	89.24%
10	489,530	139,985	71.40%	92,739	81.06%	54,117	88.95%

**Table 10:** Compression ratio of our method, WinRAR, and WinZIP.

Number	OFS	CFS-O	CR-O	CFS-RAR	CR-RAR	CFS-ZIP	CR-ZIP
1	1,166	136	88.34%	617	47.08%	676	42.02%
2	2,240	222	90.09%	887	60.40%	946	57.77%
3	6,628	887	86.62%	2,052	69.04%	2,111	68.15%
4	12,224	1,179	90.36%	3,378	72.37%	3,442	71.84%
5	22,692	2,180	90.39%	6,162	72.85%	6,150	72.90%
6	49,428	4,538	90.82%	12,504	74.70%	12,286	75.14%
7	96,994	10,416	89.26%	21,389	77.95%	21,321	78.02%
8	156,516	15,889	89.85%	34,162	78.17%	34,362	78.05%
9	269,000	28,937	89.24%	56,152	79.13%	57,671	78.56%
10	489,530	54,117	88.95%	101,269	79.31%	108,175	77.90%

**Table 11:** Compression time of four methods.

Number	File size	CT-[13]-ms	CT-[14]-ms	CT-O-ms	CT-RAR-ms
1	1,166	4	1	11	41
2	2,240	8	2	19	42
3	6,628	12	4	143	39
4	12,224	43	5	111	43
5	22,692	79	10	187	43
6	49,428	181	21	383	45
7	96,994	381	47	1,246	50
8	156,516	692	60	1,623	55
9	269,000	1,356	105	3,374	59
10	489,530	3,388	185	6,463	69

**Table 12:** Decompression time of four methods.

Number	File size	CT-[13]-ms	CT-[14]-ms	CT-O-ms	CT-RAR-ms
1	1,166	1	1	9	30
2	2,240	2	2	17	32
3	6,628	4	3	56	34
4	12,224	8	8	83	37
5	22,692	18	13	149	39
6	49,428	70	18	329	42
7	96,994	290	70	770	46
8	156,516	961	250	1,398	54
9	269,000	3,111	873	2,958	67
10	489,530	11,427	5,070	6,773	98
